# The Microbiota-gut-brain axis in vascular cognitive impairment: unraveling the mysterious link and therapeutic prospects

**DOI:** 10.3389/fimmu.2025.1648800

**Published:** 2025-10-07

**Authors:** Tingting Liu, Ying Li, Xuejiao Xiong, Xinxing Lai, Xiangqing Xu

**Affiliations:** ^1^ Department of Neurology, Affiliated Hospital of Shandong University of Traditional Chinese Medicine, Jinan, China; ^2^ First College of Clinical Medicine, Shandong University of Traditional Chinese Medicine, Jinan, China; ^3^ Institute for Brain Disorders, Beijing University of Chinese Medicine, Beijing, China; ^4^ Department of Neurology, Dongzhimen Hospital, Beijing University of Chinese Medicine, Beijing, China; ^5^ Beijing University of Chinese Medicine, Beijing, China

**Keywords:** vascular cognitive impairment, microbiota-gut-brain axis, pathogenesis, treatment, systematic review

## Abstract

**Background:**

Vascular cognitive impairment (VCI) exhibits particularly high prevalence in East Asian populations. However, its pathogenesis remains elusive due to its multifactorial and complex nature. Emerging evidence highlights the microbiota-gut-brain axis as a novel and promising paradigm for elucidating VCI mechanisms and developing therapeutic interventions. This systematic review aims to synthesize recent advances in this field, offering critical perspectives to guide future research on VCI through the lens of gut-brain interactions. Notably, given Traditional Chinese Medicine’s (TCM) holistic and multi-target therapeutic advantages, we incorporate TCM studies to complement conventional approaches.

**Methods:**

We systematically searched PubMed, EMBASE, Web of Science, Cochrane Library, China National Knowledge Infrastructure (CNKI), Chinese Science and Technology Periodical Database (VIP), and Wanfang database for relevant studies from their inception to March 31, 2025, and conducted a comprehensive review.

**Results:**

A total of 22 relevant studies were included in the final review. Current research primarily focused on analyzing the altered gut microbiota in VCI patients, with findings indicating significant changes in both the structure and abundance of gut microbiota. *Enterobacteriaceae* exhibited potential as a diagnostic biomarker for post-stroke cognitive impairment (PSCI) (AUC=0.629), while distinct microbial signatures involving *Bifidobacterium*, *Lactobacillus gasseri*, and *Anaerostipes hadrus* may effectively differentiated PSCI patients from stroke survivors without cognitive deficits (AUC values of 0.785, 0.792, and 0.750, respectively). Furthermore, multiple interventional studies from both basic and clinical research systematically explored the microbiota-gut-brain axis as a promising therapeutic target for VCI. They evaluated the efficacy of diverse approaches—such as fecal microbiota transplantation, aerobic exercise, pharmacological interventions, and acupuncture—on key outcome including gut microbiota composition, cognitive function, hippocampal integrity, and inflammatory markers. Basic experimental studies revealed that *Prevotella histicola*, *Clostridium butyricum*, aerobic exercise, and TCM improved cognitive function, whereas trimethylamine N-oxide exacerbated cognitive impairment. The efficacy of TCM was further confirmed by clinical studies.

**Conclusion:**

Research is in its early stages, but the microbiota-gut-brain axis already offers promising prospects for a deeper understanding and discovery of potential new therapeutic targets for VCI.

**Systematic Review Registration:**

https://www.crd.york.ac.uk/prospero, identifier CRD42024560293.

## 1 Introduction

Vascular cognitive impairment (VCI) refers to cognitive dysfunction caused by cerebrovascular pathologies and their risk factors, encompassing the full disease spectrum from mild cognitive impairment to dementia (1). The disorder is typically characterized by impairments in reasoning, planning, judgment and other cognitive functions, with particularly notable executive dysfunction, and may be accompanied by gait abnormalities (2). Although the exact prevalence of VCI remains undetermined, epidemiological studies show its subtype vascular dementia (VaD) ranks as the second leading cause of dementia worldwide, accounting for approximately 20-40% of all dementia cases, and appears to be the predominant dementia subtype in Southeast Asian populations (3, 4). Despite its significant clinical burden, effective therapies for VCI remain limited.

Chronic cerebral hypoperfusion serves as a primary driver of VCI. Numerous risk factors for VCI have been identified, particularly vascular risk factors including hypertension, obesity, diabetes mellitus, dyslipidemia, hyperhomocysteinemia, and smoking (5). However, the precise pathogenesis of VCI remains incompletely understood and may involve multiple interrelated mechanisms: neurovascular dysfunction, blood-brain barrier disruption, white matter damage, oxidative stress, neuroinflammation, and alterations in the gut microbiota (1, 4, 6). Among these factors, the gut microbiota has recently emerged as a crucial research focus in VCI. The gut microbiota refers to the microorganisms residing in the gastrointestinal tract, including bacteria, archaea, and eukaryotes (7). It may influence physiological, behavioral, and cognitive brain functions through the gut-brain axis via neural, immune, endocrine, and metabolic pathways (6, 8).

The gut microbiota, as a key component of the gastrointestinal tract, and emerging research suggests that the classic bidirectional interaction between the gut and the brain (brain-gut axis) should be expanded to include the gut microbiota, forming what is now termed the microbiota-gut-brain axis (9, 10). On the one hand, the brain regulates various physiological processes in the intestine through the hypothalamus-pituitary-adrenal (HPA) axis and the autonomic nervous system; on the other hand, the intestine regulates brain function through various microorganisms and their derived metabolites and products, as well as neuroactive substances. These metabolites and products pass through the enteric nervous system, vagus nerve, circulatory system and immune system to reach the brain (11, 12). Gut microbiota-derived components such as microbial antigens, cytokines, and prostaglandins can traverse the blood-brain barrier to activate the HPA axis, whereas certain microbial metabolites like short-chain fatty acids demonstrate the capacity to attenuate HPA axis responses; moreover, bacterial-derived neurotransmitters can directly interact with vagal afferent nerves (13–15). The hepatic and celiac branches of the vagus nerve exhibit particular susceptibility to stimulation by gut microbiota-derived molecules (including nitric oxide and bile acids), metabolites (such as short-chain fatty acids and trimethylamine N-oxide), and enteroendocrine hormones, which upon entering systemic circulation can exert central nervous system effects either by crossing the blood-brain barrier or via neural pathways (16–18). Through these concerted mechanisms, the gut microbiota collectively regulates neuronal activity, astrocytic function, microglial polarization states, and blood-brain barrier integrity, thereby contributing to neuroinflammatory processes, cerebrovascular dysfunction, and neuronal injury.

**Figure 1:**
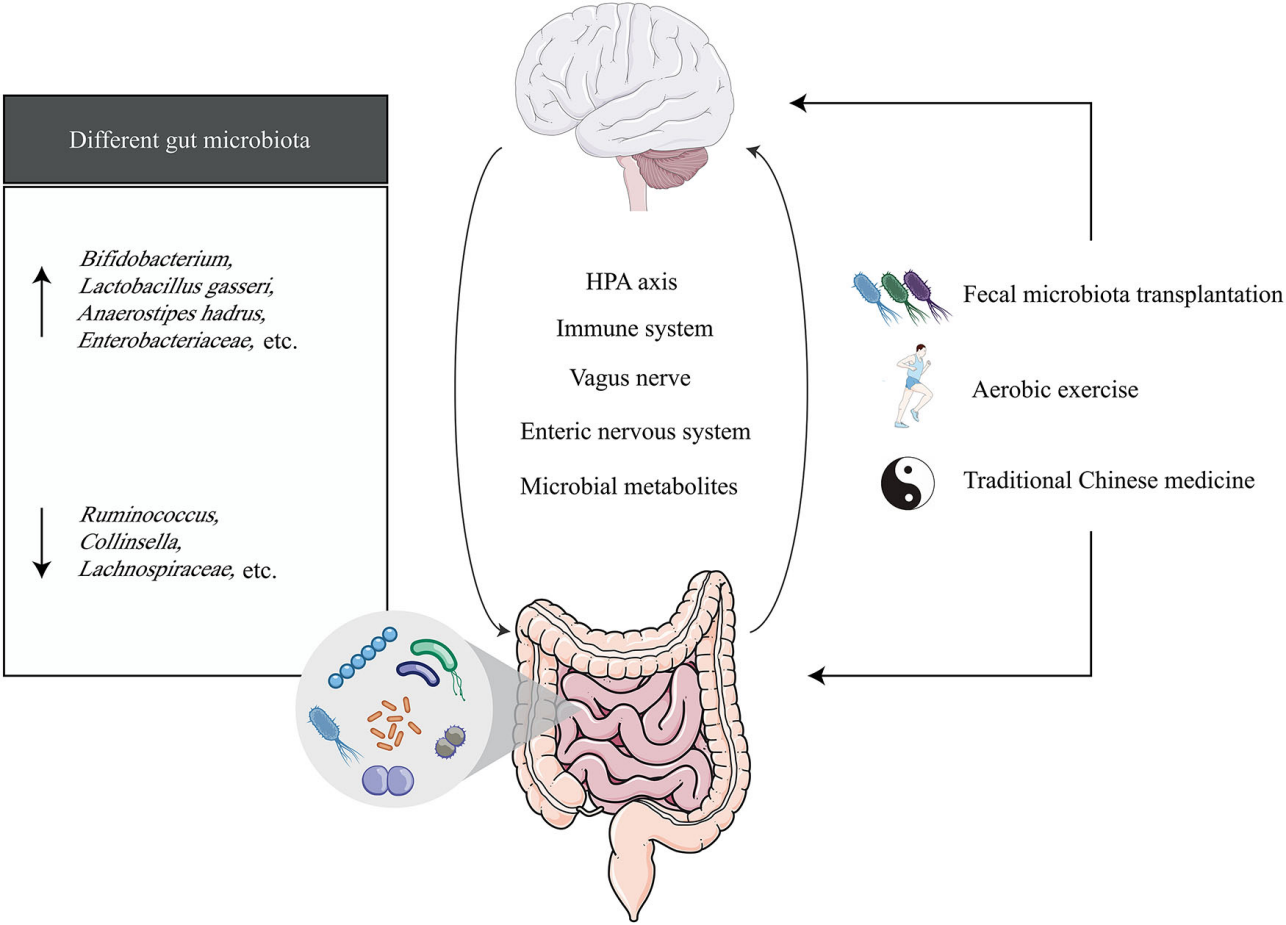
The microbiota-gut-brain axis in vascular cognitive impairment. The black arrow (↑) represents the elevated gut microbiota expressed in vascular cognitive impairment, and the black arrow (↓) represents the declining expression of the gut microbiota expressed in vascular cognitive impairment.

In recent years, a series of studies on the relationship between the microbiota-gut-brain axis and VCI have been conducted, including investigations into the characteristics of gut microbiota in VCI and the mechanisms by which the microbiota and its metabolites influence VCI progression (**Figure 1**) (19–23). The microbiota-gut-brain axis offers a promising framework for understanding and potentially treating VCI. This framework not only facilitates a deeper understanding of VCI pathogenesis but may also identify novel therapeutic targets to delay disease progression and reduce VCI risk, which is critical for advancing drug development.

Recognizing the current lack of systematic reviews addressing the relationship between the microbiota-gut-brain axis and VCI, we conducted the first systematic review to summarize recent progress and highlight the challenges that must be addressed. Furthermore, given the multifaceted pathological mechanisms underlying VCI—where conventional single-target therapies frequently demonstrate restricted clinical efficacy—our investigation intentionally incorporated TCM research due to its distinctive holistic approach and multi-target therapeutic potential. This systematic review aims to provide important insights for future research on VCI through the lens of the microbiota-gut-brain axis.

## 2 Methods

### 2.1 Search strategy and selection criteria

The study protocol was registered on PROSPERO (CRD42024560293) on June 20, 2024, and this systematic review strictly adhered to the registered protocol. Seven electronic databases were searched without language limitation (from their inception to March 31, 2025), including PubMed, EMBASE, Web of Science, Cochrane Library, China National Knowledge Infrastructure (CNKI), Chinese Science and Technology Periodical Database (VIP), and Wanfang database. All searches were performed by combining free-text and MESH terms, containing vascular cognitive impairment, vascular dementia, multiinfarct dementia, post stroke cognitive impairment, cerebral small vessel disease, brain-gut axis, gut microbiome, and intestine flora.

Two reviewers (Y.L. and X.X.) independently screened titles, abstracts and selected potential full-texts for further analysis. Those studies fulfilling our pre-defined eligibility criteria were included in the review. Any disagreements were resolved by discussion or consultation with a third reviewer (X.X.). The detailed inclusion criteria were: (a) VCI patients or VCI animals, (b) application of microbiota-gut-brain axis to study VCI, and (c) experimental or observational studies. Exclusion criteria included abstracts, editorials, letters, reviews, case reports, and review papers.

### 2.2 Data extraction

Data were independently extracted by 2 reviewers (Y.L. and X.X.) using a preformulated data collection form. A narrative summary of the results was produced according to specific data subjects, basically including: (a) the article’s author and publication year; (b) study characteristics, involving study location, VCI type, sample size, differential gut microbiota (or metabolites) and their effects, intervention methods and their effects on gut microbiota and cognitive function. For each study, all relevant data were extracted from tables, figures, text, and **Supplementary Material**.

### 2.3 Quality assessment

The risk of bias assessment for included studies was conducted independently by two reviewers (Y.L. and X.X.): randomized controlled trials were evaluated using the Cochrane Risk of Bias Tool version 2.0, while other clinical studies were assessed with the Quality Assessment Tool for Observational Cohort and Cross-Sectional Studies developed by the National Heart, Lung, and Blood Institute of the National Institutes of Health, with quality thresholds defined as ≥ 75% (good), 50-74% (fair), and < 50% (poor) (24, 25). Animal studies were evaluated using the SYRCLE’s Risk of Bias Tool (26).

## 3 Results

**Figure 2:**
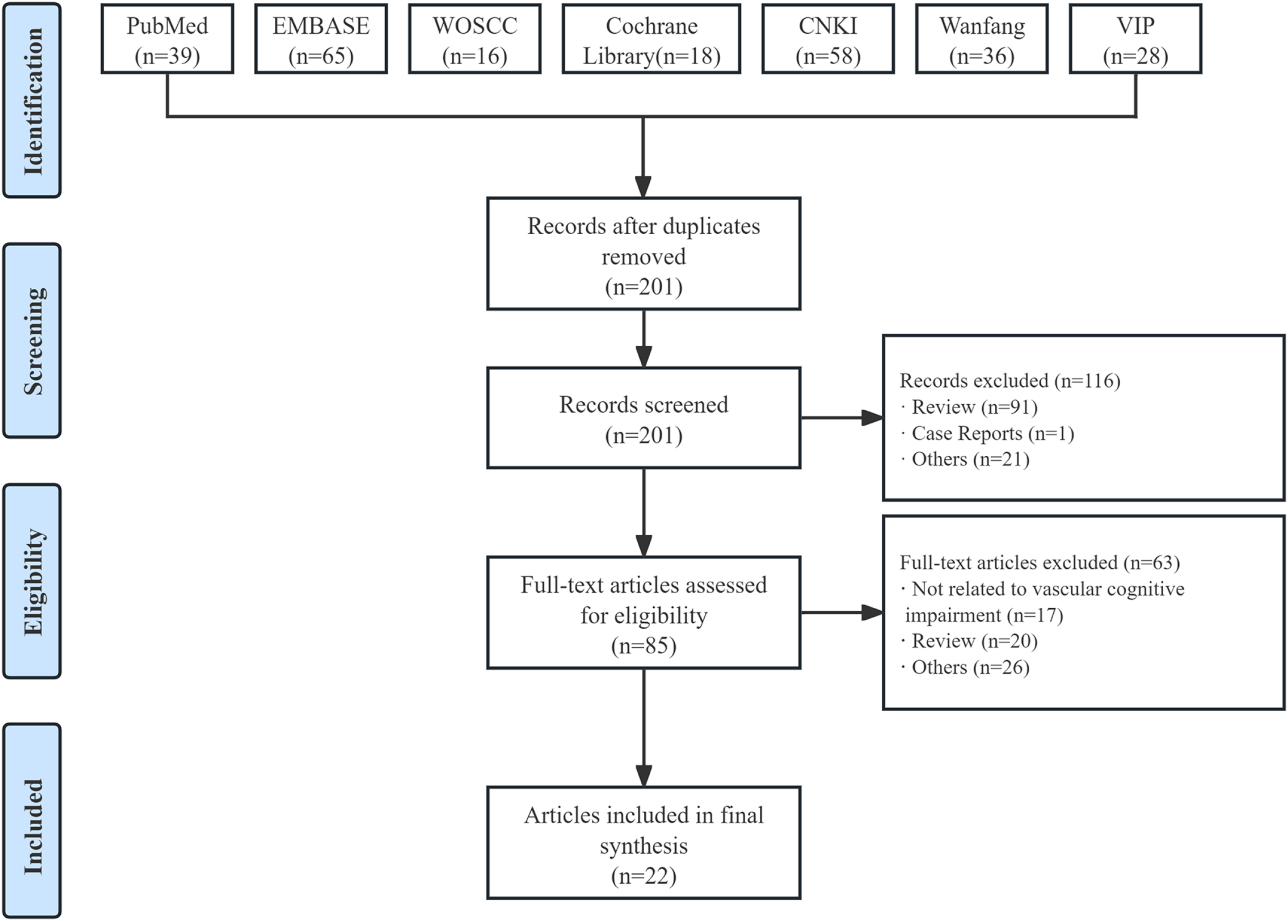
Flow diagram of study selection.

A total of 260 studies were retrieved through seven electronic database systems, with 59 excluded due to duplication. After screening titles and abstracts, 85 potentially relevant full-text articles were identified. Ultimately, 22 studies (19, 20, 22, 23, 27–44) were included in the final analysis (**Figure 2**).

Of the 22 studies on VCI, 11 were basic research experiments (20, 23, 27, 29–32, 38, 42–44), and 11 were clinical trials (19, 22, 28, 33–37, 39–41). A total of 527 rats and 129 mice were included in the basic research experiment, and a total of 787 human subjects were included in the clinical trial. In the clinical trial, there were 252 women except for two studies that did not report gender-specific data. Seven studies focused on the gut microbial characteristics of VCI, and 15 examined interventions targeting the microbiota-gut-brain axis in the context of VCI.

### 3.1 The close relationship between gut microbiota and VCI

This section included a total of seven studies, six of which analyzed the gut microbiota characteristics of VCI patients (22, 28, 33, 35, 36, 39), and one study explored the relationship between gut proteins and VCI (**Table 1**) (19). All studies demonstrated fair methodological quality (**Supplementary Table S1**).

**Table 1:** The close relationship between gut microbiota and VCI.

Source	Origin	Diseases	Population	Sample size	Sex (M/F)	Microbiome method	Gut microbiota (or metabolites) changes	Correlation
Li et al, 2022 (36)	China	PSCI	Human	PSCI: 29; stroke: 18; HC: 20	34/33	16S rRNA	↑: *Faecalibacterium*, *Bacteroides*, *Pseudomonas* ↓: *Ruminococcus*	NA
Li et al, 2022 (35)	China	PSCI	Human	PSCI: 12; PSNCI: 12; HC: 12	20/16	16S rRNA	↑: *Bifidobacterium* genus, *Alloscardovia* genus, *Alloscardovia omnilens* bacteria, *Lactobacillus gasseri* bacteria, *Anaerostipes hadrus* bacteria	*Anaerostipes hadrus* was negatively correlated with the MoCA scores; *Bifidobacterium, Lactobacillus gasseri*, and *Anaerostipes hadrus* could be used to distinguish PSCI from PSNCI patients.
Boschetti et al, 2023 (19)	Italy	VaD	Human	VaD: 18; LOAD: 40; MD: 42; aMCI: 36; HC: 23	NA	ELISA	Zonulin was not increased in VaD	NA
Ling et al, 2020 (22)	China	PSCI	Human	stroke: 93 (PSCI: 53; PSNCI: 40)	NA	16S rRNA	↑:*Gammaproteobacteria, Enterobacteriales, Enterobacteriaceae, Klebsiella, Prevotella* ↓: *Firmicutes*, and its members, including *Clostridia*, *Clostridiales*, *Lachnospiraceae*, and *Lachnospiraceae_other*	*f_Lachnospiraceae_other*, *Fusicatenibacter*, *Parasutterella*, *Phascolarctobacterium*, *Clostridium_XVIII*, and *Butyricicoccus* were positively associated with the MoCA scores; *Klebsiella*, *Enterococcus, Enterobacteriaceae_other*, *Clostridium_sensu_stricto*, *Olsenella*, *Prevotella*, *Dialister*, and *Alloprevotella* were negatively associated with the MoCA score; *Enterobacteriaceae* may be used as clinical biomarkers of PSCI.
Tian et al, 2023 (39)	China	PSCI	Human	PSCI: 19; HC: 19	19/19	16S rRNA	↑: *Lactobacillus*	*Romboutsia* and *Peptostreptococcaceae* was negatively correlated with the MoCA scores.
Xu et al, 2023 (33)	China	VaD	Human	VaD: 30; HC: 30	31/29	16S rRNA	↑: *Bacteroides*, *Lactobacillus*, *Escherichia-Shigella*, *Klebsiella*, *Prevotella̲*9, *Succinivibrionaceae*, *Enterobacteriaceae*, *Proteobacteria*	NA
Li et al, 2024 (28)	China	VCI	Human	VCI: 16; HC: 18	12/22	16S rRNA	↑: *Bifidobacterium, Veillonella*, *Ruminococcus gnavus*, *Fusobacterium*, *Erysipelatoclostridium* ↓: *Collinsella*	*Ruminococcus gnavus* was negatively associated with MoCA score, which was mediated by CBF in the bilateral hypothalamus and left amygdala.

(↑) increased; (↓) decreased; aMCI, amnestic mild cognitive impairment; AD, Alzheimer’s disease; HC, healthy control; LOAD, late-onset Alzheimer’s disease; MD, mixed dementia; MoCA, montreal cognitive assessment; NA, not applicable; PSCI, post stroke cognitive impairment; PSNCI, post stroke non cognitive impairment; VaD, vascular dementia; VCI, vascular cognitive impairment. NA, not applicable.

Four studies focused on the characteristics of gut microbiota in Chinese patients with post-stroke cognitive impairment (PSCI) and its relationship with cognitive function (22, 35, 36, 39). Through 16S rRNA sequencing of fecal samples, these studies consistently demonstrated significant structural alterations in the gut microbiome of PSCI patients. The timing of assessment varied across studies, with Li et al. and Tian et al. evaluating patients within one month post-stroke (35, 39), while Ling et al. extended the observation window to three months post-stroke (22). Notably, Li et al.’s study of 12 PSCI patients (5 females; mean age 60.75 ± 12.45 years) revealed increased abundance of *Actinobacteria* (*Bifidobacterium*, *Alloscardovia*, *Alloscardovia omnilens*) and *Firmicutes* (*Lactobacillus gasseri*, *Anaerostipes hadrus*) compared to post-stroke non-cognitive impairment (PSNCI) and control groups, with *Anaerostipes hadrus* showing a negative correlation with Montreal Cognitive Assessment (MoCA) scores (35). Tian et al.’s study of 19 PSCI patients (7 females; mean age 58.16 ± 7.81 years) demonstrated elevated *Lactobacillus* levels versus healthy controls, while *Romboutsia* and *Peptostreptococcaceae* were negatively associated with MoCA performance (39). Another study by Li et al. involving 29 PSCI patients (15 females; mean age 68.5 ± 9.9 years) observed increased conditional pathogens (*Faecalibacterium*, *Bacteroides*, *Pseudomonas*) alongside decreased beneficial bacteria like *Ruminococcus* when compared to stroke patients without cognitive impairment and healthy controls (36). Interestingly, Ling et al.’s longitudinal study of 135 ischemic stroke patients (with 93 analyzable at 3 months: 53 PSCI vs 40 PSNCI) found significantly reduced *Firmicutes* (*Clostridia*, *Clostridiales*, *Lachnospiraceae*, and *Lachnospiraceae_other*) and markedly increased *Enterobacteriaceae* abundance in the PSCI group (22). These distinct microbial patterns show promise as potential diagnostic biomarkers for PSCI monitoring and clinical management.

Similar gut microbiota alterations were observed in both VaD and VCI patients. The VaD study included 30 patients (12 females; mean age 68.17 ± 10.249 years) showing significantly increased abundances of *Bacteroides*, *Lactobacillus*, and *Escherichia-Shigella* compared to healthy controls (33). In parallel, the VCI investigation of 16 patients (9 females; mean age 69.75 ± 6.44 years) revealed elevated levels of *Bifidobacterium*, *Veillonella*, *Ruminococcus gnavus*, *Fusobacterium*, and *Erysipelatoclostridium*, alongside significantly reduced *Collinsella* abundance versus controls (28). Of particular clinical significance, *Ruminococcus gnavus* levels demonstrated a negative correlation with MoCA scores. Neuroimaging analyses further identified markedly diminished cerebral blood flow in bilateral hypothalamic and left amygdalar regions, suggesting these nutrient-sensitive brain areas may critically contribute to VCI pathogenesis through gut-brain axis interactions.

Current understanding indicated that serum zonulin plays a crucial regulatory role in maintaining intestinal and blood-brain barrier function through its modulation of tight junction proteins (45). However, emerging clinical evidence failed to demonstrate elevated zonulin levels in patients with vascular dementia (19).

### 3.2 Microbiota-gut-brain axis as a potential therapeutic target for VCI

Currently, there are no effective and therapeutic approaches for VCI. Given the well-established brain-gut connection and particularly the crucial role of gut microbiota in VCI pathogenesis, growing research attention has been directed toward targeting the microbiota-gut-brain axis as a potential treatment strategy (20, 23, 27, 29). This section includes 15 studies, comprising four clinical trials and 11 experimental research. The overall risk of bias for clinical trials was rated as moderate (**Supplementary Table S2**). With the exception of one animal study that was judged to have an unclear risk of bias, all others were assessed as high risk (**Supplementary Table S3**). These studies explored diverse intervention approaches, including gut microbiota manipulation, exercise interventions, and traditional Chinese medicine (**Table 2**).

**Table 2:** Microbiota-gut-brain axis as a potential therapeutic target for VCI.

Source	Origin	Diseases	Type of study	Population	Sample size	Sex (M/F)	Intervention	Impact on gut microbiota (or metabolites)	Impact on brain
Gut microbiota and its metabolites									
Duan et al, 2023 (20)	China	VaD	AR	Rat	2VO + *Prevotella histicola*: 30; 2VO: 30; Sham: 30	NA	*Prevotella histicola*	NA	Increased CF, synapse-associated protein expression, neurotrophic factors; Decreased the pro-inflammatory factors and glial cell-associated inflammation.
Liu et al, 2023 (29)	China	VaD	AR	Mouse	VO + CB_1_: 15; VO + CB_2_: 15; VO + CB_3_: 15; VO: 12; Sham: 12	NA	*Clostridium butyricum*	Regulated the intestinal flora; Restored butyrate levels in feces and brain.	Improved the CF; Increased the levels of BDNF and Bcl-2; Reduced the levels of Bax, neuronal apoptosis, and tissue pathological changes.
Deng et al, 2022 (27)	China	VaD	AR	Rat	2VO + TMAO: 6; 2VO + TMAO + LV-NC: 6; 2VO + TMAO + SIRT1: 6; 2VO: 6; Sham: 6; TMAO: 6	NA	TMAO	NA	Aggravated cognitive and synaptic plasticity impairment; Increased oxidative stress, apoptosis and neuroinflammation; Reduced the expression of SIRT1 protein in the hippocampu.
Zhu et al, 2021 (23)	China	PSCI	AR	Mouse	TMAO: 8; Control: 11; Choline:11	NA	TMAO	NA	Increased cerebral infarction volume; Aggravated cognitive impairment.
Exercise intervention									
Deng et al, 2022 (43)	China	VaD	AR	Rat	2VO + Swim: 12; 2VO: 12; Sham: 30	NA	Swim	↑: *Lactobacillus* ↓: *Ruminococcus* Normalized the diversity of gut microbiota and adjusted the differences between microbiota communities.	Improved the learning and memory function.
Traditional Chinese medicine									
Chen et al, 2022 (44)	China	VaD	AR	Rat	2VO + EA_1_: 10; 2VO + A_2_: 10; 2VO +EA_3_: 10; 2VO: 10; Sham: 10	NA	Electroacupuncture	↑: *Clostridiales̲unclassified* ↓: *Catabacter*, *Ruminococcus*, *Desulfovibrio*	Improved the CF; Decreased the contents of IL-1β and IL-18 in serum; Alleviated the damage to intestinal mucosa and hippocampal neurons.
Xiao et al, (40)	China	PSCI	CR	Human	PSCI + Acupuncture: 26; PSCI: 26	30/22	Acupuncture	↑: *Bifidobacterium, Lactobacillus* ↓: *Escherichia coli*	Improved CF.
Jing et al, 2024 (34)	China	PSCI	CR	Human	PSCI + Tongdu Tiaoshen acupuncture: 62; PSCI: 62	72/52	Tongdu Tiaoshen acupuncture	↑: *Bifidobacterium*, *Lactobacillus* ↓: *Escherichia coli*, *Enterococcus.*	Improved CF; Improved patients’ ability to perform activities of daily living; Increased neurotransmitter level (Ach,DA,NE,5-HT).
Xiao et al, 2024 (41)	China	PSCI	CR	Human	PSCI + Warm acupuncture: 30; PSCI + Acupuncture:30	34/26	Warm acupuncture	↑: *Bifidobacterium*, *Lactic acid* bacteria	Increased the level of GABA; Promoted brain tissue repair; Improved CF.
Jia et al, 2023 (42)	China	VaD	AR	Rat	2VO + Curcumin: 15; 2VO: 15; Sham: 15	NA	Curcumin	↑: *Robinsoniella*	Improved CF; Reduced the excessive expressions of iNOS and free radicals.
Song et al, 2023 (31)	China	VaD	AR	Rat	2VO + Baicalein-L: 10; 2VO + Baicalein-H: 10; 2VO: 10; Sham: 10	NA	Baicalein	↓: *Lactobacillus*, *Clostridium* Modulated the diversity and composition of the intestinal microbiota; Suppressed the abundance of inflammation-associated microbiota.	Improved CF; Improved chronic cerebral hypoperfusion induced inflammation in the hippocampus; Inhibited the activation of the TLR4/MyD88/NF-κB signaling pathway.
Liu et al, 2022 (32)	China	VCI	AR	Rat	2VO + Tongnao Yizhi granules: 10; 2VO + Tongnao Yizhi granules + fecal bacteria: 10; 2VO + Donepezil: 10; 2VO + fecal bacteria: 10; 2VO: 10; Sham: 10	NA	Tongnao Yizhi granules + fecal bacteria	↑: *Bacteroides*, *Actinomycetes*, the ratio of *Bacteroides*/*Firmicutes* ↓: *Firmicutes*, *Cyanobacteria*	Improved the spatial learning and memory ability.
Lu et al, 2023 (30)	China	VaD	AR	Rat	2VO + Wuzang Wenyang Huayu decoction: 13; 2VO + piracetam: 13; 2VO + Wuzang Wenyang Huayu decoction + piracetam: 13; 2VO: 13; Sham: 10; Control: 10	NA	Wuzang Wenyang Huayu decoction	27 different metabolites related to VaD.	Improved the learning and memory ability; Increased the number of hippocampal cells; Regulated whole-brain cell autophagy.
Yang et al, 2021 (37)	China	VaD	CR	Human	VaD + Tiaoshen Yizhi acupuncture combined with Dingzhi Yicong decoction: 32; VaD: 32	31/33	Tiaoshen Yizhi acupuncture combined with Dingzhi Yicong decoction	↑: *Bifidobacterium*, *Lactobacillus*, *Peptococcus*, *Saccharomycetes*, *Bacteroides* ↓: *Enterococcus*, *Bacillus coli*, *Clostridium parvum*	Improved the mental state, CF, social capability, daily life function.
Duan et al, 2024 (38)	China	VaD	AR	Rat	2VO + Shibing Xingnao granule: 10; 2VO + Tongdu Tiaoshen acupuncture: 10; 2VO + Shibing Xingnao + Tongdu Tiaoshen: 10; 2VO: 10; Sham: 10	NA	Shibing Xingnao granule combined with Tongdu Tiaoshen acupuncture	Increased the relative abundance of probiotic bacteria in the intestine.	Improved the CF; Reduced the expression of Caspase-3, Bax and Bcl-2 proteins in hippocampus.

(↑) increased; (↓) decreased; Ach, acetyl choline; AR, Animal research; BNDF, brain-derived neurotrophic factor; CB, *Clostridium butyricum*; CF, cognitive function; CR, clinical research; DA, dopamine; EA, Electroacupuncture; GABA, γ-aminobutyric acid; 5-HT, 5-hydroxytryptamine; iNOS, inducible nitric oxide synthase; LV, lentivirus; NC, negative control; NE, noradrenaline; PSCI, post stroke cognitive impairment; SIRT1, silent information regulator 1; TMAO, Trimethylamine N-Oxide; VCI, vascular cognitive impairment; VaD, vascular dementia; VO, carotid artery occlusion. NA, not applicable.

#### 3.2.1 Gut microbiota and its metabolites

*Prevotella histicola*, an obligate anaerobic species within the *Prevotella* genus, demonstrated significant neuroprotective effects in a rat model of vascular dementia induced by bilateral common carotid artery ligation. Thirty 8-week-old rats receiving six-week oral administration of *Prevotella histicola* showed improved cognitive performance compared to the VaD model group, as evidenced by reduced escape latency and increased target quadrant duration in Morris water maze tests. Additionally, the treatment modulated multiple molecular markers, including upregulated synaptic proteins (MAP2, SYP, PSD-95) and downregulated pro-inflammatory cytokines (IL-1β, TNF-α, IL-6) (20).

*Clostridium butyricum*, a natural commensal organism in the gastrointestinal tracts of healthy humans and animals, has well-documented gastrointestinal benefits supported by extensive *in vivo* and *in vitro* studies (46). In a study using a murine vascular dementia model established by unilateral carotid artery occlusion, six-week administration of *Clostridium butyricum* induced multidimensional neuroprotective effects in 15 six-week-old mice compared to the VaD model group. Specifically, the treated mice exhibited enhanced spatial learning ability in behavioral tests alongside alleviated cognitive impairment. At the histological and molecular levels, the treatment improved hippocampal morphology and regulated apoptotic signaling pathways—evidenced by increased BDNF and Bcl-2 expression, decreased Bax levels, and enhanced Akt phosphorylation—thereby attenuating neuronal apoptosis. Furthermore, the therapy restored gut microbial homeostasis and normalized butyrate concentrations in both fecal samples and brain tissue brain tissue (29).

Furthermore, two independent studies investigated the role of trimethylamine N-oxide (TMAO), a gut microbiota-derived metabolite generated from dietary choline, betaine, and carnitine (23, 27). The first study demonstrated that compared to normal diet controls, three-week choline supplementation prior to stroke induction in mice significantly elevated plasma TMAO levels, exacerbating both cerebral infarct volume and cognitive impairment as evidenced by impaired performance in Y-maze and Barnes maze tests (23). In the second study, rats received TMAO administration beginning four weeks prior to VaD model induction and continuing for four weeks post-surgery, starting from the second postoperative day. Compared to the VaD model group, TMAO-treated animals exhibited more severe cognitive deficits as demonstrated by Morris water maze testing, along with aggravated oxidative stress markers, neuroinflammation (NLRP3 inflammasome activation), and neuronal apoptosis (27). Mechanistically, TMAO treatment activated the NLRP3 inflammasome while suppressing hippocampal expression of silent information regulator 1 (SIRT1).

#### 3.2.2 Exercise intervention

Aerobic exercise training has demonstrated efficacy in enhancing aerobic endurance, promoting vascular health, and improving quality of life, while simultaneously repairing neurovascular damage induced by ischemia and modulating synaptic plasticity (47–49). Emerging evidence further indicates that exercise exerts beneficial effects on gut microbiota metabolic function, augmenting microbial diversity and reinforcing the microbiota-gut-brain axis (50, 51).

In a VaD rat model induced by permanent bilateral common carotid artery occlusion, a four-week aerobic exercise intervention consisting of daily 20-minute non-loaded swimming sessions was implemented post-model establishment (43). Exercise intervention induced a substantial remodeling of gut microbial composition, characterized by a significant reduction in *Ruminococcus* abundance concomitant with increased *Lactobacillus* colonization. These microbial changes were paralleled by marked enhancements in cognitive function, as evidenced by improved learning and memory performance in behavioral assessments. Furthermore, the aerobic exercise effectively restored gut microbial diversity and induced significant modifications in inter-community microbiota structure, suggesting a comprehensive exercise-mediated modulation of the gut ecosystem in the vascular dementia model.

#### 3.2.3 Traditional Chinese medicine

Given the complex pathological mechanisms underlying VCI, conventional single-target therapeutic strategies often demonstrate limited clinical efficacy. In contrast, TCM employs a holistic, multi-target approach, positioning it as a potentially valuable intervention for VCI prevention and management (52, 53). Current TCM research on VCI primarily focuses on acupuncture and herbal therapies. This section systematically reviews 10 studies evaluating various TCM modalities, including acupuncture, moxibustion, herbal formulations, and combined acupuncture-herbal regimens.

(i)Acupuncture

Acupuncture, a well-established external therapy in TCM, demonstrates notable safety, reliability, and clinical efficacy. By stimulating specific acupoints, this intervention achieves therapeutic effects while exhibiting significantly fewer adverse reactions compared to conventional pharmaceutical treatments (54, 55).

Experimental studies utilizing a vascular dementia rat model demonstrated that electroacupuncture stimulation at key acupoints (Baihui [GV20], Dazhui [GV14], Shenshu [BL23], and Zusanli [ST36]) produced significant therapeutic effects (44). The treatment modulated gut microbiota composition by increasing *Clostridiales̲unclassified* abundance while reducing *Catabacter*, *Ruminococcus*, and *Desulfovibrio* populations. Furthermore, electroacupuncture intervention decreased serum levels of pro-inflammatory cytokines IL-1β and IL-18, protected against intestinal mucosal and hippocampal neuronal damage, and ameliorated cognitive dysfunction in the animal model.

Clinical investigations involving PSCI patients across three studies consistently demonstrated the therapeutic benefits of acupuncture interventions (34, 40, 41). The first trial employed a combined approach of acupuncture (targeting Fengfu [DU16], Dazhui [GV14], Shendao [DU11], Baihui [GV20], and Shenting [DU24]) with cognitive training, resulting in enhanced cognitive scale performance, elevated serum neurotransmitter levels (acetylcholine, dopamine, norepinephrine, and 5-hydroxytryptamine), and favorable gut microbiota alterations characterized by increased *Bifidobacterium* and *Lactobacillus* populations alongside decreased *Escherichia coli* and *Enterococcus* abundance (34). A second study incorporating warm needle therapy at Zhongwan (RN12), Tianshu (ST25), Zusanli (ST36), and Shangjuxu (ST37) with cognitive rehabilitation showed increased *Bifidobacteria* and *Lactobacilli* counts, elevated plasma γ-aminobutyric acid (GABA) concentrations, and associated cognitive improvements. A third study applied scalp acupuncture combined with body acupuncture to treat PSCI patients using the same acupoints: Zhongwan (RN12), Tianshu (ST25), Zusanli (ST36), and Shangjuxu (ST37). The results indicated that after treatment, the number of *Bifidobacteria* and *Lactobacilli* increased significantly, cognitive function improved, and the abundance of *Escherichia coli* decreased (40).

(ii)Chinese herbs

Both curcumin, a bioactive phenolic compound from *Curcuma longa* (turmeric), and baicalein, a key flavonoid in *Scutellaria baicalensis* roots, demonstrate potent anti-inflammatory, antioxidant, and free radical-scavenging properties (56–58). Experimental studies in vascular dementia models revealed distinct neuroprotective mechanisms: curcumin administration significantly increased *Robinsoniella* abundance while reducing inducible nitric oxide synthase (iNOS) expression and oxidative stress markers (free radicals and hydroxyl radicals), ultimately improving memory function (42); whereas baicalein treatment modulated gut microbiota by decreasing *Lactobacillus* and *Clostridium* populations, while concurrently attenuating neuroinflammation through reduced glial activation, suppressed proinflammatory cytokine release, and inhibition of the TLR4/MyD88/NF-κB pathway, accompanied by preserved CA1 hippocampal neuronal integrity and enhanced cognitive performance (31).

(iii)Chinese herbal compound

Wuzang Wenyang Huayu decoction, composed of Ganjiang (*Zingiberis rhizoma*), Fuzi (*Aconiti radix lateralis praeparata*), Guizhi (*Cinnamomi ramulus*), Bajitian (*Morindae officinalis radix*), Yinyanghuo (*Epimedii herba*), Banxia (*Pinelliae rhizoma*), Sanqi (*Notoginseng radix et rhizoma*), Shichangpu (*Acori tatarinowii rhizoma*), and Dahuang (*Rhei radix et rhizoma*), was found to improve learning and memory abilities in rats with vascular dementia (30). Mechanistic studies revealed this formulation promoted hippocampal neurogenesis, suppressed Caspase-3-mediated apoptosis, and enhanced autophagic activity through upregulation of APG5L/ATG5, Beclin-1, and LC3A/B protein expression. Fecal metabolomic analysis identified 27 dementia-associated metabolites, with pathway enrichment analysis implicating vitamin B metabolism, purine metabolism, pyrimidine metabolism, and histidine metabolic pathways.

Tongnao Yizhi granules, composed of Dahuang (*Rhei radix et rhizoma*), Yujin (*Curcumae radix*), Huangqi (*Astragali radix*), Chuanxiong (*Chuanxiong rhizoma*), Shichangpu (*Acori tatarinowii rhizoma*), Gouqi (*Lycii fructus*), Shuizhi (*Hirudo*), Yizhiren (*Alpiniae oxyphyllae fructus*), and Xianhecao (*Agrimoniae herba*), were combined with fecal bacteria capsules prepared from the fresh feces of healthy rats (32). This treatment significantly altered gut microbial ecology, characterized by increased of *Bacteroides* abundance, elevated *Bacteroides/Firmicutes* ratio, and enriched *Actinomycetes* populations in VCI rats, while reducing the abundance of *Firmicutes* and *Cyanobacteria*. The intervention improved spatial learning and memory abilities in behavioral tests.

(iv)Combination of acupuncture and Chinese medicine

The Tongdu Tiaoshen acupuncture protocol involved acupoints such as Shenting (DU24), Baihui (GV20), Dazhui (DU14), Zhiyang (DU9), and Yaoyangguan (DU3). Shibing Xingnao granules, composed of Huangqi (*Astragali radix*), Shichangpu (*Acori tatarinowii rhizoma*), Yuanzhi (*Polygalae radix*), Chuanxiong (*Chuanxiong rhizoma*), and Bingpian (*Borneolum Syntheticum*), were combined with acupuncture in vascular dementia rats (38). This integrated treatment demonstrated behavioral improvements through reduced escape latency and enhanced cognitive function, while also exerting neuroprotective effects via increased Bcl-2 expression and decreased Caspase-3/Bax protein levels. Furthermore, the intervention modulated gut microbiota composition by promoting beneficial bacterial populations.

The Tiaoshen Yizhi acupuncture method targeted acupoints including Shenting (DU24), Sishencong (EX-HN1), Renzhong (DU26), Neiguan (PC6), Daling (PC7), Rangu (KI2), Xuehai (SP10), and Taichong (LR03). Dingzhi Yicong formula was composed of Dangshen (*Codonopsis radix*), Shichangpu (*Acori tatarinowii rhizoma*), Yuanzhi (*Polygalae radix*), Yizhiren (*Alpiniae oxyphyllae fructus*), Danggui (*Angelicae sinensis radix*), Shudihuang (*Rehmanniae radix*), Chishao (*Paeoniae radix rubra*), Fuling (*Poria*), Gouqi (*Lycii fructus*), Chuanxiong (*Chuanxiong rhizoma*), Taoren (*Persicae semen*), and Honghua (*Carthami flos*). These two methods were combined to treat vascular dementia patients (37). The combined therapy improved cognitive performance and restored gut microbial balance, as evidenced by increased abundance of B*Bifidobacterium*, *Lactobacillus*, *Peptococcus*, *Saccharomycetes*, and *Bacteroides*, alongside decreased levels of *Enterococcus*, *Escherichia coli*, and *Clostridium parvum*.

## 4 Discussion

This systematic review provides the first comprehensive synthesis of the microbiota-gut-brain axis in VCI, encompassing both pathophysiological mechanisms and therapeutic interventions. Our analysis of 22 studies reveal several key findings that advance our understanding of VCI pathogenesis and highlight promising treatment approaches targeting gut-brain interactions.

The observed gut microbiota dysbiosis across VCI subtypes provides robust evidence for the involvement of the microbiota-gut-brain axis in disease pathogenesis. The identification of specific microbial signatures, particularly the increased abundance of *Enterobacteriaceae* in PSCI, suggests its potential as diagnostic biomarkers. Furthermore, the negative correlations observed between certain bacterial taxa (e.g., *Anaerostipes hadrus* and *Ruminococcus gnavus*) and cognitive performance underscore the functional significance of these microbial alterations.

The reviewed studies primarily focus on three principal intervention strategies targeting the gut-brain axis in VCI management. First, direct modulation of gut microbiota through probiotic administration or fecal microbiota transplantation has demonstrated significant neuroprotective effects, including cognitive improvement, neuroinflammation reduction, and synaptic plasticity enhancement. Second, aerobic exercise has emerged as an effective modulator of gut-brain interactions, demonstrating the capacity to restore microbial diversity, mitigate pro-inflammatory cytokines, and enhance cognitive outcomes. Third, TCM approaches, incorporating both acupuncture and herbal formulations, exhibit multi-target effects on gut microbiota composition, inflammatory markers, and neuronal protection, reflecting the potential advantages of holistic interventions for addressing VCI’s complex pathophysiology. The consistent observations of improved gut barrier function and reduced inflammation across various intervention modalities further substantiate the critical role of gut-brain communication in VCI.

The current evidence also reveals important gaps and limitations in the field. Although differential gut microbiota and their metabolites have been preliminarily identified, the research remains exploratory and requires large-scale validation. More importantly, the precise mechanistic relationships between specific microbial alterations and VCI pathogenesis need further elucidation, as only by clarifying these specific mechanisms can more targeted therapeutic strategies be developed. Furthermore, existing studies exhibit significant heterogeneity in study design, including variations in cognitive impairment severity and methodological differences in microbial analysis, which complicate direct comparisons across studies.

In future studies, first, it is essential to implement a well-designed study framework and adopt standardized inclusion and exclusion criteria as much as possible to facilitate the integration and analysis of results across different studies. For studies with relatively small sample sizes, strict matching of variables such as age, gender, and underlying diseases is essential to minimize the influence of confounding factors. Alternatively, consideration should be given to conducting large-scale prospective cohort studies similar to the “Determinants of Incident Stroke Cognitive Outcomes and Vascular Effects on RecoverY (DISCOVERY) study” (59). Currently, a multicenter cohort study evaluating the predictive value of gut microbiota and serum biomarkers for cognitive impairment and poor prognosis after ischemic stroke is underway (NCT:04688138, ClinicalTrials.gov). Second, all differential gut microbiota identified must undergo external validation before clinical applications, which will help objectively identify VCI and simplify diagnostic procedures. Third, more in-depth mechanistic research on the discovered differential gut microbiota is necessary to lay the foundation for developing effective VCI treatment strategies. Fourth, therapeutic interventions such as aerobic exercise, microbiota transplantation, and TCM still need to be further evaluated in standardized, well-designed clinical trials. Probiotics also represent a treatment approach targeting, with an ongoing clinical trial evaluating the efficacy of Bifidobacterium lactis Probio-M8 for post-stroke cognitive impairment (ChiCTR2400079870, Chinese Clinical Trial Registry). Fifth, to ensure data quality, strict standard operating procedures must be established for sample collection, preservation, processing, and testing (60). The standardization of these procedures is crucial for maintaining consistency and accuracy across studies. Additionally, future research should incorporate neuroimaging assessments. As an indispensable tool in VCI research, neuroimaging not only reveals pathophysiological mechanisms but also evaluates intervention effects, demonstrating increasingly prominent value in both clinical and research applications (61).

## 5 Conclusion

This systematic review establishes the microbiota-gut-brain axis as a critical framework for understanding VCI pathogenesis and developing novel treatment strategies. Although characteristic alterations in gut microbiota composition and abundance have been consistently documented in VCI patients, these findings require further validation. Furthermore, the mechanistic interplay between gut microbial dysbiosis, microbial-derived metabolites, and VCI pathobiology remains to be fully delineated, representing a crucial knowledge gap that demands systematic investigation through integrated multi-omics approaches and longitudinal study designs.

## Data Availability

The original contributions presented in the study are included in the article/**Supplementary Material**. Further inquiries can be directed to the corresponding authors.
